# Supplementary material for: Infection characteristics among Serratia marcescens capsule lineages
Source: mBio. 2025 Apr 16;16(5):e00559-25. doi: 10.1128/mbio.00559-25 (PMC12077157; doi:10.1128/mbio.00559-25)
Supplement: Fig. S1 — Polysaccharide phenotypes of capsule mutant strains. [file mbio.00559-25-s0001.pdf]

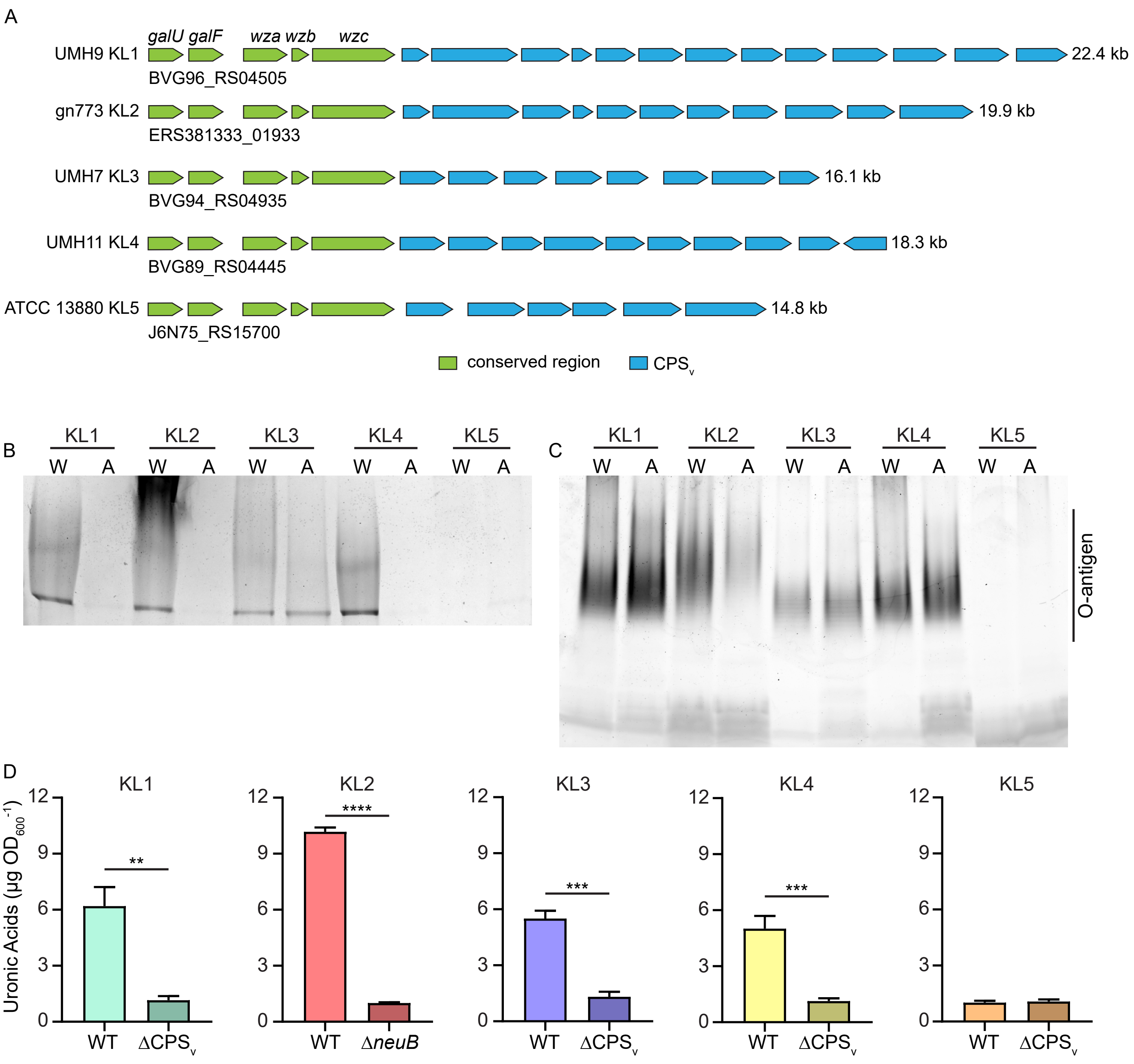

Supplemental Figure 1. Polysaccharide phenotypes of capsule mutant strains. A. Genetic organization of the five KL relevant to this study. The locus tag of the first open reading frame (arrows) (*galU*) in each KL is provided for reference. B. Total polysaccharides isolated from wild-type cells (W) and acapsular mutant derivatives (A) were separated by SDS-PAGE and stained with alcian blue. C. The same polysaccharides from panel A were electrophoresed with a 7-20% SDS-PAGE gel and stained with ProQ Emerald green to visualize LPS components. D. Measurement of extracellular uronic acids in wild-type (WT) and capsule mutant derivatives of KL1-5 strains. Statistical significance was assessed by unpaired t-test: **, $P < 0.01$; ***, $P < 0.001$; ****, $P < 0.0001$.
