# Supplementary material for: Infection characteristics among Serratia marcescens capsule lineages
Source: mBio. 2025 Apr 16;16(5):e00559-25. doi: 10.1128/mbio.00559-25 (PMC12077157; doi:10.1128/mbio.00559-25)
Supplement: Fig. S3 — Serum exposure does not induce ATCC 13880 capsule synthesis. [file mbio.00559-25-s0003.pdf]

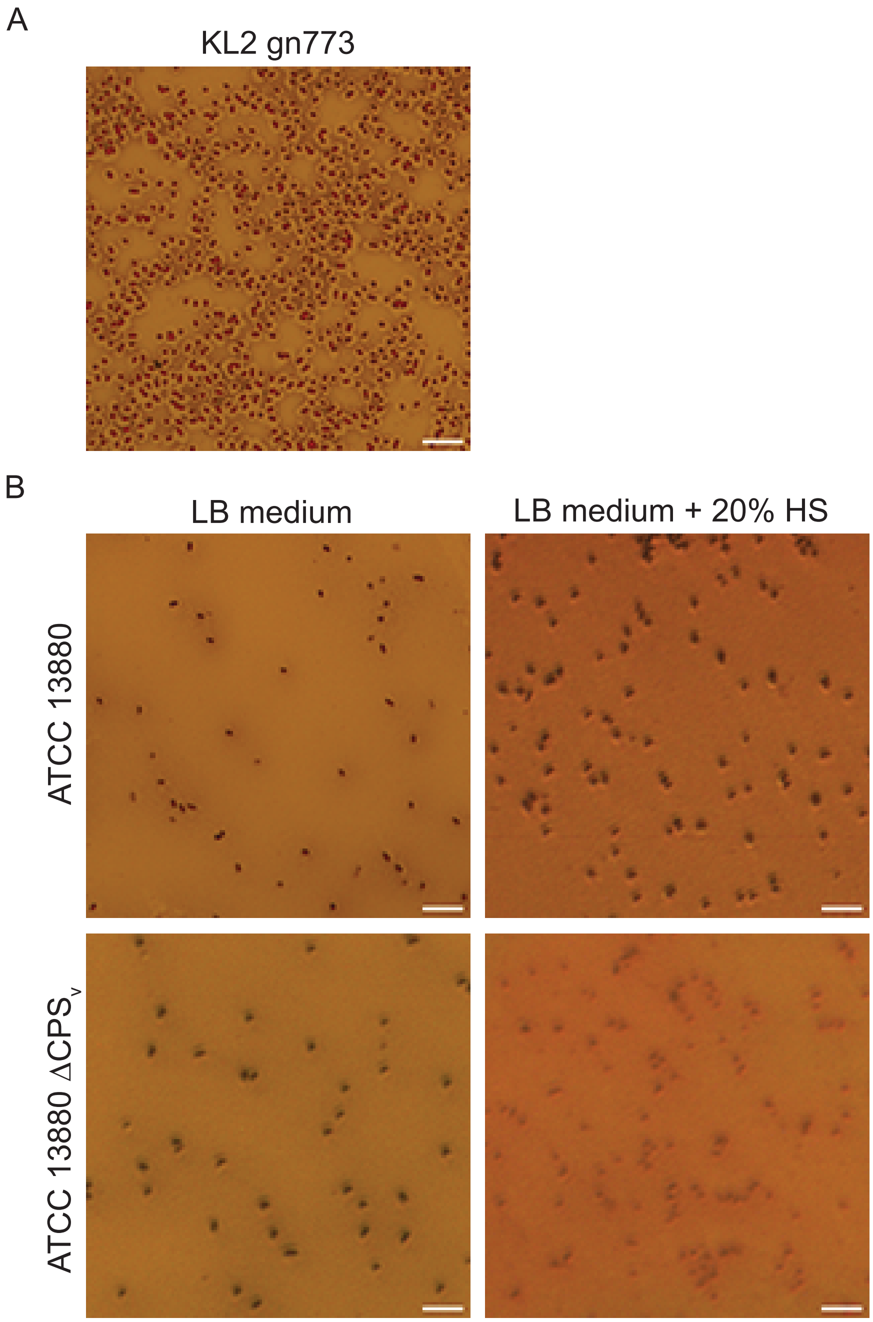

Supplemental Figure 3. Serum exposure does not induce ATCC 13880 capsule synthesis. A. A control culture of encapsulated strain KL2 gn773 was stained with Maneval's reagent, demonstrating a negative staining region surrounding gn773 cells indicative of capsule. B. ATCC 13880 and the ΔCPS_v derivative were passaged in increasing concentrations of human serum (HS) over the course of three days and subjected to Maneval stain after the final passage in 20% HS. No evidence of CPS synthesis was detected from either strain by this method. Scale bars are 5 μm.
