# Supplementary material for: Infection characteristics among Serratia marcescens capsule lineages
Source: mBio. 2025 Apr 16;16(5):e00559-25. doi: 10.1128/mbio.00559-25 (PMC12077157; doi:10.1128/mbio.00559-25)
Supplement: Fig. S4 — CPS production by non-clinical isolates. [file mbio.00559-25-s0004.pdf]

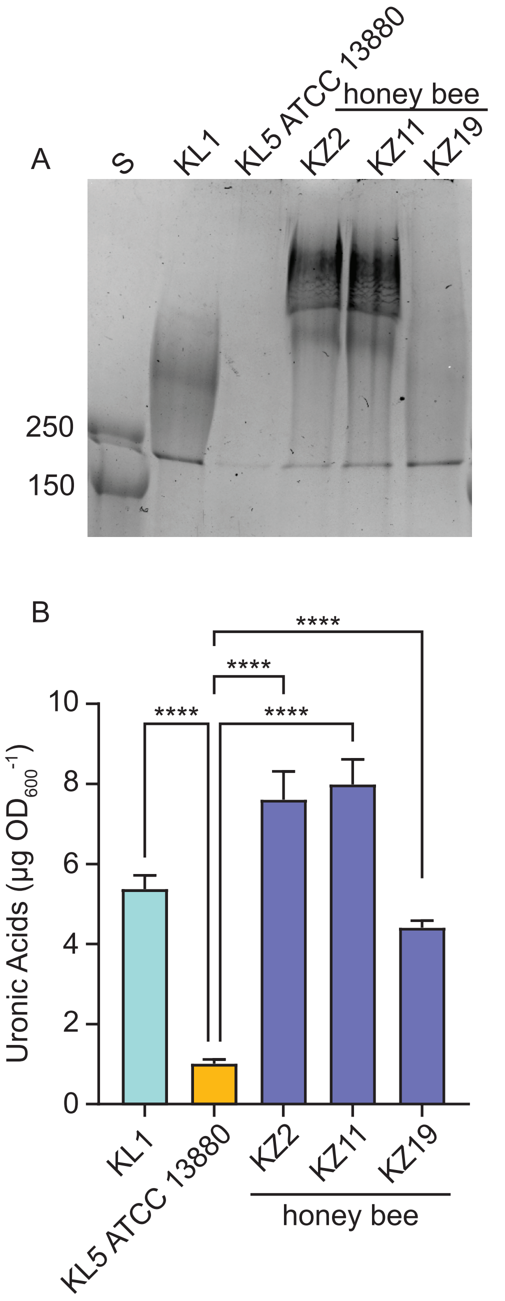

Supplemental Figure 4. CPS production by non-clinical isolates. A. Total polysaccharides were isolated from the indicated *S. marcescens* strains and separated by SDS-PAGE in comparison to protein standards (S) of known molecular weight (kDa). Gels were stained with alcian blue for visualization of CPS. B. Quantitation of extracellular uronic acids in *S. marcescens* strains. Statistical significance was assessed by one-way ANOVA with Dunnetts multiple comparisons test against ATCC 13880; ****, P < 0.0001.
